# Cytotoxic lymphocyte heterogeneity in glioblastoma: insights from single-cell and spatial multiomics toward precision immunotherapeutic reprogramming

**DOI:** 10.3389/fimmu.2026.1837084

**Published:** 2026-06-10

**Authors:** Wentao Dong, Yang Li, Zhuolin Wu, Quanlin Fu, Jinshuang Zhao, Bangyue Wang, Jia Yao, Chenglu Lu, Minfeng Tong

**Affiliations:** 1 Department of Neurosurgery, Affiliated Jinhua Hospital, Zhejiang University School of Medicine, Jinhua, Zhejiang, China; 2 Department of Neurosurgery, Tianjin Medical University General Hospital, Tianjin, Tianjin, China; 3 Tianjin Medical University, Tianjin, Tianjin, China; 4 Department of Oncology, Tianjin Huanghe Hospital, Tianjin, Tianjin, China; 5 Department of Neurosurgery, the Second Affiliated Hospital of Anhui Medical University, Hefei, Anhui, China; 6 Department of Radiology, Tianjin Medical University General Hospital, Tianjin, Tianjin, China; 7 Department of Pathology, Hebei Key Laboratory of Molecular Oncology, Tangshan People’s Hospital, Tangshan, China

**Keywords:** blood-brain barrier, cytotoxic lymphocytes, glioblastoma, immunotherapy, NK cell dysfunction, progenitor exhausted T cells, single-cell transcriptomics, spatial genomics

## Abstract

Glioblastoma (GBM) represents the most aggressive primary brain tumor in adults, characterized by a profoundly immunosuppressive tumor microenvironment (TME) that systematically disables cytotoxic lymphocyte function and renders conventional immunotherapy largely ineffective. While exhaustion of CD8^+^ T cells and natural killer (NK) cells within solid tumors has been extensively studied in other cancer types, the CNS-specific architectural, metabolic, and molecular constraints that shape cytotoxic lymphocyte heterogeneity in GBM remain insufficiently characterized. Recent advances in single-cell RNA sequencing (scRNA-seq) and spatial multiomics have begun to reveal a rich landscape of cytotoxic lymphocyte subpopulations in GBM. These include TCF-1^+^ progenitor-exhausted T cells (Tpex), terminally exhausted CD8^+^ T cells (Tex), and dysfunctional natural killer (NK) cell subsets, each distributed across anatomically distinct immune niches. This review synthesizes current knowledge across three interconnected areas: the single-cell atlas of GBM-infiltrating cytotoxic lymphocytes; the spatial organization of their dysfunction within perinecrotic, perivascular, and infiltrative-edge niches; and the epigenetic and transcriptional programs that underlie GBM-specific cytotoxic failure, including dysregulation of the TOX/TCF-1 axis and IDH-mutation-driven silencing of NKG2D ligands. Critically, we compare CD8^+^ T cell and NK cell exhaustion mechanisms, highlighting their mechanistic divergence and therapeutic implications. We further discuss how these multiomics insights can be translated into neurosurgically relevant strategies, including intraoperative tumor profiling, progenitor T cell expansion via epigenetic priming, NKG2A/TIGIT dual blockade, and intracavitary delivery of engineered NK cells. Together, this review proposes a spatially and cellularly resolved framework for understanding cytotoxic immune failure in GBM and outlines precision immunotherapy approaches tailored to the unique immunobiology of the CNS tumor microenvironment.

## 1 Introduction

Glioblastoma (WHO Grade 4) is the most common and lethal primary brain tumor in adults, with a median overall survival of approximately 15 months despite maximal surgical resection, radiotherapy, and temozolomide chemotherapy (1). The profound resistance of GBM to current therapies—including immune checkpoint inhibitors (ICIs) that have revolutionized outcomes in other solid tumors—stems in large part from its uniquely immunosuppressive tumor microenvironment (2, 3). Unlike most solid tumors, GBM resides within the central nervous system (CNS), a site of relative immune privilege, where the blood-brain barrier (BBB), restricted lymphatic drainage, abundant immunosuppressive tumor-associated macrophages (TAMs), and local TGF-β/IDO signaling converge to disable adaptive and innate immune responses (4).

Cytotoxic lymphocytes—principally CD8^+^ T cells and natural killer (NK) cells—represent the primary effectors of antitumor immunity and the theoretical targets of immunotherapy. In GBM, these cells infiltrate the tumor parenchyma but are rapidly rendered dysfunctional through a process of chronic stimulation, receptor-ligand imbalance, and transcriptional reprogramming that collectively constitute ‘exhaustion’ (5, 6). Critically, the biology of cytotoxic lymphocyte exhaustion in GBM is not merely an amplified version of that observed in peripheral solid tumors: the CNS microenvironment imposes unique spatial, metabolic, and epigenetic constraints that must be specifically understood before rational immunotherapy can be designed.

The advent of single-cell RNA sequencing, spatially resolved transcriptomics (e.g., 10x Visium, Xenium, MERFISH), and integrative multiomics approaches has created an unprecedented opportunity to dissect the heterogeneity of cytotoxic lymphocytes in GBM at single-cell resolution and with spatial context preserved (7, 8). Recent scRNA-seq studies have identified discrete CD8^+^ T cell subpopulations within GBM—including TCF-1^+^ progenitor-exhausted (Tpex) and terminally exhausted (Tex) subsets—as well as NK cell subpopulations exhibiting a stress-associated, maturation-impaired ‘exhaustion-like’ phenotype distinct from T cell exhaustion (9, 10). Spatial profiling has further revealed that these populations occupy anatomically segregated immune niches within the tumor, with profound implications for their functional state and therapeutic accessibility.

Several recent reviews have addressed GBM immunotherapy, tumor microenvironmental heterogeneity, and single-cell or spatial transcriptomic profiling of gliomas. The distinctive contribution of the present review is not to claim first coverage of these topics, but to provide a cytotoxic-lymphocyte-centered framework that integrates CD8^+^ Tpex/Tex dynamics and NK-cell dysfunction with GBM-specific spatial niches, molecular suppressive drivers, neurosurgical sampling considerations, local delivery strategies, and immunotherapeutic reprogramming approaches. In doing so, we aim to synthesize how single-cell and spatial multiomics can inform a critical and translationally relevant model of cytotoxic immune failure in GBM, while explicitly acknowledging the methodological limitations and cohort-dependent nature of current evidence.

## 2 The CNS-specific immunosuppressive landscape of GBM

### 2.1 Structural barriers to cytotoxic lymphocyte infiltration

The CNS poses unique structural barriers to immune surveillance that profoundly limit the entry and accumulation of cytotoxic lymphocytes within GBM tumors. The blood-brain barrier (BBB) is composed of tight-junction-linked endothelial cells, astrocytic endfeet, and pericytes. It restricts lymphocyte transmigration by downregulating adhesion molecules (ICAM-1 and VCAM-1) and disrupting the chemokine gradients that normally facilitate T cell trafficking (11). In GBM, despite partial BBB disruption within the tumor core, the invasive edge—where diffusely infiltrating tumor cells reside—retains substantial barrier integrity, creating a ‘lymphocyte-excluded’ zone that corresponds precisely to the region of greatest surgical concern for recurrence (12).

NK cells, which do not require antigen-specific priming for activation, possess distinct CNS entry mechanisms. CX3CR1^+^ NK cells can traverse the choroid plexus and meningeal borders utilizing CX3CL1/fractalkine gradients, and a subset of NK cells has been detected in the perivascular space of GBM tumors (13). However, the absolute numbers of NK cells within GBM parenchyma remain low, and those that do infiltrate face rapid functional suppression by the intratumoral microenvironment (14). CD8^+^ T cells, by contrast, appear to require CXCR3/CXCL10 signaling for GBM infiltration, a pathway that is frequently downregulated in GBM TME secondary to TGF-β-mediated suppression of CXCL10 production by astrocytes and myeloid cells.

### 2.2 The immunosuppressive cellular ecosystem

GBM is distinguished from most solid tumors by the extreme dominance of myeloid cells—comprising microglia and bone marrow-derived tumor-associated macrophages (TAMs)—within the TME, which can represent up to 50% of the total tumor mass (15). These TAMs adopt predominantly immunosuppressive (M2-like) phenotypes under the influence of GBM-derived signaling molecules, including IL-10, TGF-β, CSF-1, and CCL2. In turn, they secrete IL-10, arginase-1 (Arg-1), and indoleamine 2,3-dioxygenase (IDO), which collectively impair the metabolic fitness of infiltrating cytotoxic lymphocytes (16). Spatial transcriptomics data from GBM patient cohorts have revealed that immunosuppressive HMOX1^+^ TAMs spatially co-localize with terminally exhausted CD8^+^ T cells in the perinecrotic niche, establishing a self-reinforcing suppressive ‘ecosystem’ (17).

Beyond myeloid cells, GBM also recruits regulatory T cells (Tregs) and myeloid-derived suppressor cells (MDSCs) that further amplify local immune suppression. The resulting cytotoxic lymphocyte landscape is paradoxically characterized by both quantitative scarcity (low infiltration) and qualitative dysfunction (high exhaustion burden), presenting a dual therapeutic challenge fundamentally different from ‘immunologically hot’ tumors such as melanoma or microsatellite-instable colorectal cancer (18).

### 2.3 The paradox of cytotoxic lymphocyte scarcity and dysfunction

A critical but underappreciated feature of GBM immunobiology is that even the limited CD8^+^ T cells and NK cells that do penetrate the tumor demonstrate a disproportionately high burden of functional exhaustion markers (PD-1, TIM-3, LAG-3, TIGIT) relative to other tumor types (9, 19). This suggests that the CNS microenvironment does not merely exclude cytotoxic cells but actively accelerates their differentiation toward terminal exhaustion once they do infiltrate. Several CNS-specific factors contribute to this acceleration. First, GBM is characterized by elevated interstitial pressure, which activates mechanosensory pathways—including Piezo1 and its downstream target Osr2—that promote terminal T cell differentiation. Second, immunosuppressive metabolites such as kynurenine, 2-hydroxyglutarate (2-HG; in IDH-mutant cases), and lactate accumulate at high concentrations within the tumor microenvironment. Third, the intratumoral tertiary lymphoid structures (TLS) that support progenitor-exhausted T cell (Tpex) maintenance in other cancers are largely absent in GBM (20, 21). These features establish GBM as a paradigmatic model of cytotoxic immune failure driven by CNS-intrinsic constraints.

An additional layer of complexity is malignant tumor-cell-state heterogeneity. GBM is not composed of a single malignant cell program. Single-cell studies have shown that GBM cells can adopt at least four distinct transcriptional states: oligodendrocyte progenitor-like (OPC-like), neural progenitor-like (NPC-like), astrocyte-like (AC-like), and mesenchymal-like (MES-like). The relative abundance of these states varies across patients, genetic backgrounds, and tumor regions (22). These malignant-cell states may differentially shape cytotoxic lymphocyte distribution and function by altering chemokine gradients, antigen-presentation context, vascular organization, metabolic stress, myeloid recruitment, and NK-activating ligand expression. Therefore, CD8^+^ T cell exhaustion and NK cell dysfunction in GBM should not be interpreted as immune-intrinsic processes alone, but as outcomes of bidirectional interactions between cytotoxic lymphocytes, suppressive myeloid cells, and spatially heterogeneous tumor-cell programs.

Among these malignant programs, MES-like tumor states are particularly relevant to immune remodeling. Macrophage-derived oncostatin M has been shown to induce MES-like transitions in GBM cells through OSMR/LIFR–GP130–STAT3 signaling, illustrating that immune cells can actively reshape malignant-cell states rather than merely respond to them (23). Conversely, MES-like, hypoxic, or perinecrotic tumor regions may reinforce myeloid-dominant suppression, metabolic stress, and cytotoxic lymphocyte dysfunction. Spatial transcriptomic and anatomic transcriptional studies further indicate that GBM molecular programs vary across histologically and radiographically distinct compartments, including cellular tumor core, perinecrotic zones, microvascular regions, and infiltrative edges (24, 25). Thus, cytotoxic lymphocyte scarcity and dysfunction should be interpreted together with malignant-cell-state architecture, especially in spatial analyses that aim to link immune cell states to anatomical niches and therapeutic accessibility.

Beyond the cellular and spatial determinants of immune suppression outlined above, cytotoxic lymphocytes infiltrating GBM must simultaneously contend with a hostile metabolic landscape. Immunometabolic stress represents an additional and mechanistically distinct dimension of cytotoxic failure in this tumor.

### 2.4 Immunometabolic constraints on cytotoxic lymphocytes in GBM

Immunometabolic stress is a central determinant of cytotoxic lymphocyte dysfunction in GBM. In addition to inhibitory receptor signaling and myeloid-derived suppression, CD8^+^ T cells and NK cells must operate within a metabolically hostile tumor microenvironment characterized by glucose competition, hypoxia, lactate accumulation, tryptophan catabolism, lipid remodeling, and mitochondrial stress. Tumor cells can restrict T cell glycolysis and mTOR activity by competing for glucose, thereby reducing IFN-γ production and effector function (26). In GBM, this metabolic pressure is reinforced by two overlapping mechanisms. Glycolytic monocyte-derived macrophages undergo glucose-driven histone lactylation, which promotes their immunosuppressive activity. Concurrently, tumor-derived lactate drives immune evasion through histone lactylation and upregulation of the anti-phagocytic signal CD47 (27, 28). Thus, glucose and lactate metabolism may suppress cytotoxic lymphocytes directly by limiting their metabolic fitness and indirectly by reinforcing suppressive macrophage and tumor-cell programs.

Mitochondrial dysfunction further links the GBM microenvironment to cytotoxic failure. Tumor-infiltrating CD8^+^ T cells can undergo loss of mitochondrial mass and function, reduced PGC(Peroxisome proliferator-activated receptor-gamma coactivator)-1α-dependent mitochondrial biogenesis, and impaired effector activity within solid tumors (29). In GBM, hypoxia, chronic antigen exposure, lactate accumulation, TGF-β signaling, and nutrient limitation may similarly compromise mitochondrial fitness in Tpex-like, transitional, and Tex-like CD8^+^ T cells. Amino acid metabolism also contributes to immune suppression: IDO-mediated tryptophan depletion and kynurenine accumulation can impair T cell function, while kynurenine can reduce NK-cell activating receptors such as NKp46 and NKG2D (30). In IDH-mutant gliomas, 2-HG-driven epigenetic remodeling further links tumor oncometabolism to NK-cell immune evasion through suppression of NKG2D ligand expression.

Lipid and cholesterol metabolism should also be considered when interpreting cytotoxic lymphocyte dysfunction in GBM. Although cholesterol is required for membrane organization and immune synapse formation, excessive cholesterol accumulation in the tumor microenvironment can promote CD8^+^ T cell exhaustion through ER stress and XBP1-dependent induction of inhibitory receptors (31). For NK cells, metabolic dysfunction is closely tied to tumor-induced reprogramming. GBM stem cells impair NK-cell function through an αv integrin/TGF-β signaling axis. Blocking this pathway—or engineering NK cells to lack the TGF-β receptor (TGFBR2)—restores NK-cell activity against GBM stem cells in preclinical models (32). Together, these findings indicate that cytotoxic lymphocyte failure in GBM is not solely a receptor-driven exhaustion process, but also a metabolically constrained state. Future therapeutic strategies should therefore consider spatially and cell-type-specific metabolic interventions, including lactate modulation, mitochondrial protection, IDO/kynurenine targeting, cholesterol/ER-stress control, and engineering of T or NK cells to resist TGF-β- and lactate-rich niches.

Collectively, the structural, cellular, and metabolic features described in this section establish GBM as a profoundly hostile environment for cytotoxic lymphocyte function. However, understanding these bulk-level suppressors is insufficient for therapeutic design: cytotoxic lymphocytes in GBM are not a uniform population but rather a heterogeneous collection of functional states, each with distinct persistence, exhaustion trajectories, and therapeutic vulnerabilities. Single-cell transcriptomic and multiomics approaches have made it possible to resolve this heterogeneity, and Section 2 provides a comprehensive atlas of CD8^+^ T cell and NK cell subpopulations within the GBM microenvironment.

## 3 Single-cell atlas of cytotoxic lymphocytes in GBM

### 3.1 CD8^+^ T cell subpopulations revealed by scRNA-seq

#### 3.1.1 Heterogeneity and exhaustion trajectory

Single-cell transcriptomic studies of GBM-infiltrating CD8^+^ T cells have consistently identified multiple functional subpopulations arrayed along a spectrum from early activation to terminal exhaustion (9, 33). The canonical framework distinguishes four populations. The first is TCF-1^+^PD-1^+^ progenitor-exhausted T cells (Tpex; also denoted CD8T TCF7^+^), which retain self-renewal capacity, respond to PD-1 blockade, and represent the principal “resource pool” for anti-tumor immunity. The second is an intermediate exhausted population (Tint; CD8T GZMK^+^) with partial effector function and migratory capacity. The third is terminally exhausted T cells (Tex; CD8T GZMB^+^ LAG3^+^HAVCR2^+^TCF7^-^), which exhibit high cytotoxic gene expression but irreversible dysfunction. The fourth is a CD8^+^ TEMRA-like effector population (CD8T GZMB^+^CX3CR1^+^), associated with a circulating rather than tissue-resident phenotype (33, 34).

These scRNA-seq-derived labels are best understood as operational cell-state annotations whose boundaries vary across datasets, platforms, and annotation strategies (see Section 2.3 for detailed caveats).

**Figure 1:**
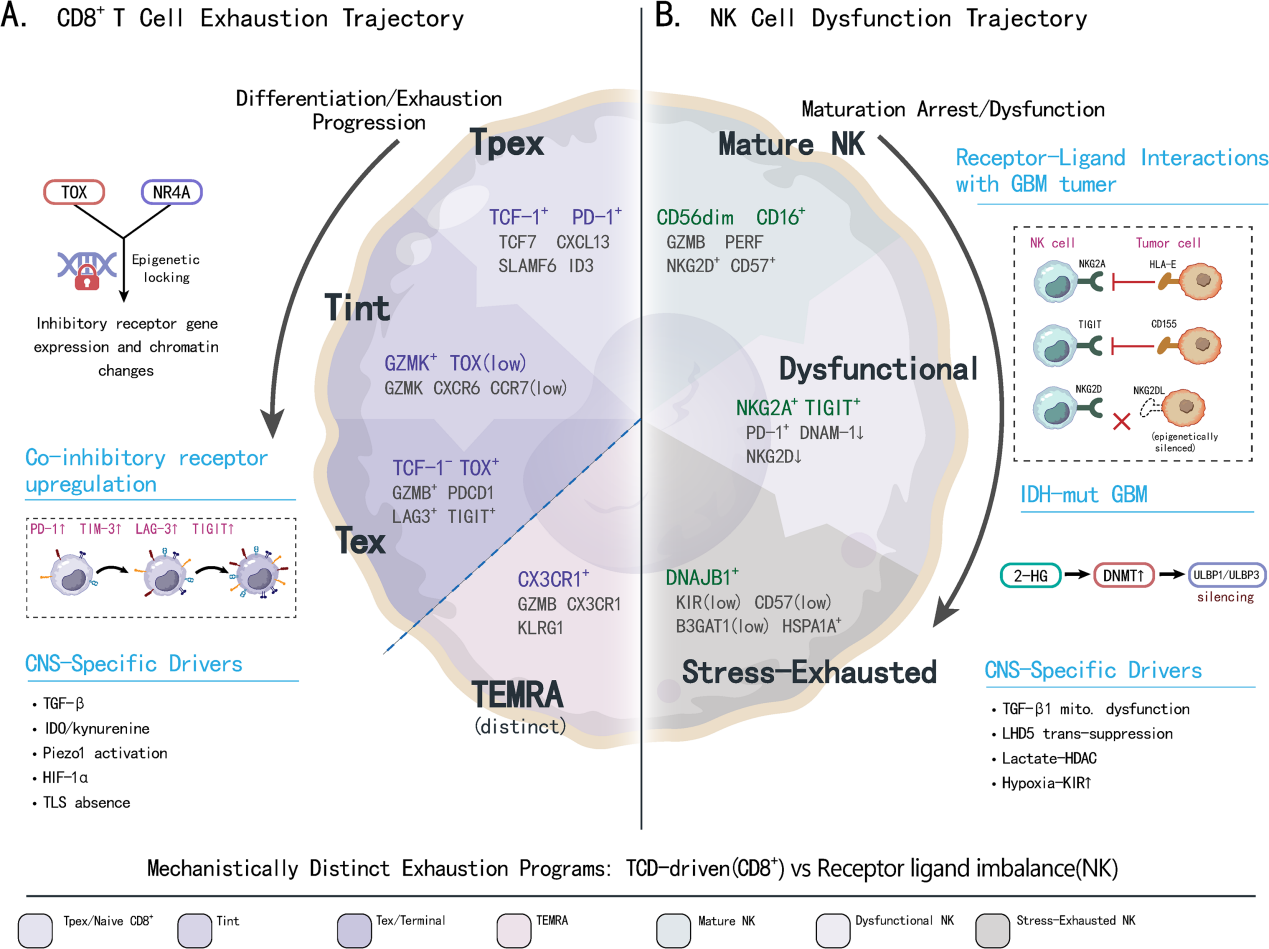
CD8^+^T Cell and NK Cell Exhaustion Trajectories in GBM: A Dual-Pathway Model. Dual exhaustion trajectories of CD8^+^ cytotoxic T cells and NK cells in the glioblastoma tumor microenvironment. **(A)** CD8^+^ T cell differentiation spectrum from progenitor-exhausted (Tpex; TCF-1^+^ PD-1^+^ SLAMF6^+^) through intermediate-exhausted (Tint; GZMK^+^ TOXlow) to terminally exhausted (Tex; GZMB^+^ TOX^+^ LAG-3^+^ TIGIT^+^ TCF-1^-^) states, and a distinct CX3CR1^+^ TEMRA effector population. Progressive upregulation of co-inhibitory receptors (PD-1, TIM-3, LAG-3, TIGIT) is indicated. TOX/NR4A-mediated epigenetic locking of the terminally exhausted state is highlighted. CNS-specific drivers annotated: Piezo1, TGF-β, IDO, TLS absence. **(B)** NK cell trajectory from mature cytotoxic (CD56dim CD16^+^ NKG2D^+^) through dysfunctional (NKG2A^+^ TIGIT^+^ NKG2D↓) to stress-exhausted (DNAJB1^+^ KIRlow) states. Inhibitory receptor–ligand axes (NKG2A/HLA-E; TIGIT/CD155; NKG2D/NKG2DL) depicted. IDH-mutant GBM NKG2DL silencing specifically annotated. Mechanistic divergence between CD8^+^ T cell exhaustion (TCR-driven, TOX/NR4A) and NK cell dysfunction (receptor–ligand imbalance, ligand silencing) is emphasized. Tpex, progenitor-exhausted T cell; Tint, intermediate-exhausted; Tex, terminally exhausted; TEMRA, effector memory re-expressing CD45RA; 2-HG, 2-hydroxyglutarate; IDH, isocitrate dehydrogenase; TLS, tertiary lymphoid structure.

A pivotal 2024 study in Nature Communications, drawing on multiple GBM and low-grade glioma scRNA-seq datasets, confirmed that exhausted CD8^+^ T cells in brain tumors co-express a program of inhibitory molecules including PDCD1 (PD-1), TIGIT, TOX, and LAG3. Complementary TCR sequencing revealed that the highest-frequency T cell clonotype belonged to this exhausted population—indicating that clonal expansion continues even as functional capacity is progressively lost (9). Critically, high-grade gliomas (HGGs) harbored significantly fewer Tpex cells relative to Tex cells compared to low-grade gliomas (LGGs), suggesting that the transition from progenitor to terminal exhaustion is accelerated in the more aggressive GBM TME. Quantitatively, Tpex cells (TCF7^+^CD8^+^) typically comprise 5–15% of total CD8^+^ TILs in GBM scRNA-seq datasets, while terminally exhausted Tex cells (GZMB^+^LAG3^+^TCF7^-^) account for 40–60% of intratumoral CD8^+^ T cells in HGG specimens, yielding a Tpex: Tex ratio approximately 2–3-fold lower in HGG than in LGG (9). **Figure 1** demonstrates the CD8^+^ T cell and NK cell exhaustion trajectories in GBM. It delineates the lineage trajectory of cell subsets and alterations in receptor expression.

At the same time, this pattern should not be generalized as a uniform property of every GBM specimen. Inter-patient variability, IDH status, primary versus recurrent disease, corticosteroid exposure, prior radiochemotherapy, and sampling from the tumor core versus infiltrative margin may all influence the observed Tpex: Tex ratio. Therefore, GBM CD8^+^ T cell exhaustion is best considered a context-dependent trajectory in which progenitor-like, intermediate, terminally exhausted, and effector-like states may coexist in different proportions across cohorts.

Because the Tpex and Tex annotations used in GBM scRNA-seq studies derive from a broader literature on chronic antigen stimulation, chronic viral infection, and extracranial tumors, a brief conceptual clarification of Tpex biology is necessary before discussing GBM-specific constraints.

#### 3.1.2 Canonical biology and context-dependent heterogeneity of Tpex cells

The concept of progenitor-exhausted CD8^+^ T cells was established by studies showing that chronic antigen exposure does not generate a uniformly terminally dysfunctional CD8^+^ T cell population. Instead, a TCF7/TCF-1-associated progenitor-like subset persists during chronic stimulation, maintains self-renewal capacity, and continuously supplies more differentiated exhausted progeny. In this review, Tpex refers to progenitor-, precursor-, stem-like, or memory-like exhausted CD8^+^ T cells, whereas Tex refers to terminally exhausted CD8^+^ T cells with high inhibitory receptor expression, reduced proliferative capacity, and limited reversibility. Developmentally, Tpex cells generally arise early during chronic antigen stimulation, before terminal exhaustion becomes transcriptionally and epigenetically fixed, and persist as an upstream reservoir that replenishes downstream exhausted or effector-like populations.

Foundational studies in chronic viral infection demonstrated that Tpex-like biology is linked to both transcriptional programming and anatomical localization. He et al. identified virus-specific CXCR5^+^CD8^+^ T cells during chronic LCMV(Lymphocytic choriomeningitis virus) infection that migrated into B-cell follicles. Compared with CXCR5^-^ cells, these cells expressed lower levels of PD-1 and Tim-3, showed stronger cytokine production and cytotoxicity, and were regulated by the Id2-E2A transcriptional axis (35). Importantly, CXCR5^+^CD8^+^ T cells could further differentiate into CXCR5^-^ progeny during chronic infection, supporting the concept that CXCR5^+^ Tpex-like cells can function as an upstream progenitor-like reservoir rather than a terminally differentiated population. Leong et al. further described CXCR5^+^ follicular cytotoxic T cells that entered B-cell follicles to eliminate infected T follicular helper cells(TFH) and B cells; their differentiation required Bcl6, E2A, and TCF-1, while Blimp1, Id2, and Id3 restrained this program (36). These studies established that chronic antigen exposure can generate spatially specialized CD8^+^ T cell states with progenitor-like and follicular-positioning features rather than a single terminally exhausted population.

Tumor studies subsequently translated this concept to the intratumoral setting. Siddiqui et al. showed that mouse and human tumors contain Tcf1^+^PD-1^+^CD8^+^ TILs with stem-like properties, including expansion, self-renewal, and differentiation into Tcf1^-^PD-1^+^ progeny after therapeutic vaccination or checkpoint blockade (37). Thus, PD-1 expression alone does not define terminal exhaustion, because Tcf1^+^PD-1^+^ cells can retain proliferative and regenerative capacity despite expressing an inhibitory receptor. Importantly, ablation of these Tcf1^+^ tumor-reactive T cells impaired tumor control, indicating that immunotherapy relies on expansion of a stem-like intratumoral pool rather than simple reversal of terminal exhaustion. This study also showed that intratumoral Tcf1^+^PD-1^+^ T cells can self-renew within the tumor microenvironment and sustain the production of differentiated Tcf1^-^PD-1^+^ progeny even when new lymphocyte influx is restricted, supporting a local intratumoral progenitor–progeny relationship.Importantly, ablation of these Tcf1^+^ tumor-reactive T cells impaired tumor control, indicating that immunotherapy relies on expansion of a stem-like intratumoral pool rather than simple reversal of terminal exhaustion. Jansen et al. further showed that human tumors contain clonally related TCF1^+^TIM3^-^CD28^+^ stem-like CD8^+^ T cells and checkpoint-high terminally differentiated progeny, with the stem-like cells residing in dense MHC-II^+^ antigen-presenting-cell niches (38). Loss of such APC-rich niches was associated with poor CD8^+^ T cell infiltration and disease progression, highlighting the importance of local niche support for maintaining the Tpex reservoir.This distinction is particularly relevant for GBM, because a tumor may lack fully organized tertiary lymphoid structures yet still contain APC-rich or perivascular immune neighborhoods capable of supporting Tpex-like cells.

Clinical single-cell studies in melanoma provide additional evidence that TCF7^+^ progenitor-like CD8^+^ T cell states are linked to checkpoint inhibitor responsiveness. Sade-Feldman et al. identified a TCF7^+^IL7R^+^ memory-like CD8 G state enriched in responding melanoma lesions, whereas CD39^+^TIM3^+^ exhausted CD8 B cells were enriched in non-responding lesions. The TCF7^+^CD8^+^/TCF7^-^CD8^+^ ratio predicted anti-PD-1 response and was associated with longer survival. However, this study also illustrates context dependence: CXCR5 is prominent in chronic infection-associated follicular cytotoxic CD8^+^ T cells but is not a universal marker of TCF7^+^ tumor-infiltrating CD8^+^ T cells (39). Fine clustering in melanoma further revealed multiple memory-like, effector-like, transitional, proliferative, and exhausted-like CD8^+^ T cell states, suggesting that Tpex-like cells and their downstream progeny exist along a continuum rather than as two rigid compartments.

Together, these studies indicate that not all Tpex cells are created equal. Tpex-like cells may differ in CXCR5 expression, APC niche dependence, anatomical localization, proliferative potential, cytotoxic priming, and responsiveness to checkpoint blockade. Their division of labor may include maintaining an antigen-experienced progenitor reservoir, generating intermediate or terminally exhausted progeny, providing the proliferative burst after PD-1 blockade, and coordinating local antitumor immunity with APC-rich niches. Within this hierarchy, Tpex-like cells primarily preserve self-renewal and proliferative competence; intermediate or GZMK^+^ transitional cells may retain partial effector and migratory capacity; and terminal Tex cells may execute cytotoxic programs but have limited proliferative recovery. Therefore, immune checkpoint blockade appears to depend most strongly on mobilization of the progenitor-like Tpex pool, whereas fully terminal Tex cells may be less reversible because of fixed transcriptional and epigenetic remodeling.

In GBM, TCF7^+^PDCD1^+^ cells should be evaluated by integrated transcriptional, clonal, spatial, and functional criteria—not assumed to be identical to Tpex states in melanoma, NSCLC, or chronic viral infection—given the myeloid-dominant, TLS-poor, and metabolically constrained GBM microenvironment. The analytical caveats relevant to cross-study comparison of these populations are discussed in Section 2.3.

#### 3.1.3 The Tpex–Tex axis: GBM-specific constraints

The relative depletion of Tpex in GBM compared with other solid tumors has profound therapeutic implications. Available glioma and GBM datasets consistently show an unfavorable Tpex: Tex balance and insufficient Tpex-supportive niche structure relative to ICI-responsive tumors, though the magnitude of this imbalance varies across cohorts and is subject to the analytical caveats discussed in Section 2.3.

In peripheral tumors such as melanoma and NSCLC, Tpex cells—identified by co-expression of TCF-1, PD-1, and CXCL13—form the cellular substrate for responses to anti-PD-1 therapy; in melanoma, a high TCF7^+^CD8^+^/TCF7^-^CD8^+^ ratio was associated with significantly improved progression-free survival (HR ~0.4) and objective response to anti-PD-1 blockade, while the TCF7^+^ fraction comprised 30–50% of CD8^+^ TILs in responding lesions (39–41). In GBM, the Tpex pool is constrained by at least three CNS-specific factors. First, intratumoral tertiary lymphoid structures (TLS)—which provide the CXCL13-rich niche required for Tpex maintenance in other tumors—are largely absent. Second, the elevated interstitial pressure characteristic of GBM activates Piezo1-mediated mechanotransduction, which drives TCF-1 downregulation and accelerates the premature transition from Tpex to terminally exhausted T cells (Tex). Third, IDO-mediated tryptophan depletion suppresses the Wnt/β-catenin signaling required to maintain TCF-1 expression (20, 42).

Evidence from non-CNS tumors demonstrates that Tpex-like cells can be sustained not only within classical TLSs but also in APC-rich intratumoral immune neighborhoods. In GBM, perivascular immune regions therefore represent candidate Tpex-supportive niches—a hypothesis that should be tested by spatial transcriptomics, multiplexed protein imaging, and paired TCR tracking, as discussed further in Section 3.3.

These data suggest that strategies to expand or preserve the Tpex pool—rather than simply blocking inhibitory checkpoints—may be particularly critical in GBM. As discussed in Section 6, epigenetic priming agents such as decitabine can restore TCF-1^+^ Tpex proportions in preclinical GBM models, opening a potential therapeutic avenue (43).

Current evidence supports epigenetic priming as a means to preserve or expand progenitor-exhausted CD8^+^ T cell states in preclinical models; whether the primary mechanism in GBM involves peripheral expansion or local intratumoral maintenance of Tpex-like cells remains to be resolved through paired blood–tumor TCR sequencing and spatial pharmacodynamic profiling.

### 3.2 NK cell subpopulations and exhaustion-like states

#### 3.2.1 NK cell scRNA-seq Atlas in GBM

NK cells in GBM are substantially less studied than CD8^+^ T cells at the single-cell level, yet emerging data reveal a similarly complex and dysfunction-prone landscape. scRNA-seq analyses of GBM-infiltrating NK cells have identified several subpopulations, including: (1) CD56bright NK cells enriched for cytokine production (IFN-γ, TNF-α) but low cytotoxicity; (2) CD56dim CD16^+^ mature NK cells with high granzyme B/perforin expression but marked upregulation of inhibitory receptors (NKG2A, TIGIT, PD-1); and (3) a DNAJB1^+^ CD56dim ‘stress-exhausted’ subset with heat-shock protein signatures, low KIR family expression, low CD57 (indicating immaturity), and enriched PD-L1/PD-1 pathway genes—resembling the terminally exhausted T cell state in its transcriptional profile but arising through distinct mechanisms (13, 44). A key mechanistic distinction between NK cell and T cell exhaustion in GBM lies in their induction pathways. T cell exhaustion is driven primarily by chronic T cell receptor (TCR) stimulation, which triggers TOX- and NR4A-mediated epigenetic “locking” of the exhausted state. NK cell dysfunction, by contrast, arises through at least three distinct mechanisms. The first is chronic engagement of inhibitory receptors—including NKG2A/HLA-E, TIGIT/CD155, and KIR/MHC-I—without sufficient compensatory activating signals. The second is epigenetic silencing of activating ligands on tumor cells, which deprives NK cells of the stimulatory cues needed for activation (discussed further in Section 5.2). The third is TGF-β1-induced mitochondrial dysfunction, which impairs granzyme B exocytosis and thereby blunts cytotoxic capacity (14, 45). This mechanistic divergence has direct implications for therapeutic targeting, as approaches effective for T cell reinvigoration (anti-PD-1) may not fully restore NK cell cytotoxicity in GBM.

NK cell “licensing” (also termed education) is the developmental process by which NK cells acquire functional competence through sustained engagement of inhibitory receptors—principally KIR family members and the NKG2A/CD94 heterodimer—with cognate self-MHC class I molecules during maturation (46). Licensed NK cells exhibit heightened responsiveness to activating stimuli, whereas unlicensed NK cells remain hyporesponsive. This paradigm has critical implications for GBM immune evasion: while classical HLA-A/B/C downregulation on GBM cells would theoretically trigger “missing self” recognition by licensed NK cells, GBM simultaneously overexpresses the non-classical molecule HLA-E, which engages NKG2A on licensed NK cells and delivers a dominant inhibitory override that suppresses cytotoxicity (47, 48). This dual strategy—shedding classical MHC-I to evade T cell surveillance while substituting a specialized ligand that specifically disables the most potent licensed NK subset—represents a GBM-intrinsic immune evasion architecture in which the two main cytotoxic lymphocyte populations are suppressed through molecularly distinct but spatially convergent mechanisms.

Although current GBM single-cell studies primarily emphasize dysfunctional or exhaustion-like NK cell states, evidence from non-GBM systems suggests that NK cells may also contain TCF-1-associated memory-like or stem-like subsets. Kujur et al. demonstrated that Zika virus infection induces CD27^+^ memory-like NK cells with stem cell-like properties, including expansion capacity, self-renewal-associated pathways, differentiation into effector NK cells, longer telomeres, hematopoietic stem/progenitor-associated gene signatures, active Wnt/β-catenin signaling, and high TCF-1 expression. These cells, termed “NK memory stem cells,” exhibited greater antiviral potential than naïve or non-memory NK cells after adoptive transfer (49). In parallel, Dogra et al. provided a human multi-tissue NK cell atlas showing that tissue localization shapes NK cell development, maturation, function, and residence, with precursor or immature NK populations enriched in lymphoid and mucosal tissues and mature or terminally differentiated NK cells predominating in blood, bone marrow, spleen, and lung (27). These findings broaden the conceptual framework for interpreting NK cell heterogeneity in GBM: in addition to identifying stress-exhausted or inhibitory receptor-high NK states, future GBM scRNA-seq and spatial studies should determine whether TCF7/TCF-1^+^ stem-like or memory-like NK reservoirs exist within the tumor, whether they localize to specific perivascular or lymphoid-like niches, and whether they can be therapeutically expanded. However, because the current evidence for TCF-1^+^ stem-like NK cells derives mainly from viral infection models and human tissue atlases rather than GBM, this concept should be regarded as a testable hypothesis rather than an established feature of the GBM immune microenvironment.

#### 3.2.2 IDH mutation-specific NK cell evasion

A GBM-specific dimension of NK cell dysfunction is provided by IDH mutation status. IDH-mutant gliomas—while less frequently classified as GBM under the 2021 WHO classification—share the GBM TME in the context of IDH-mutant GBM (IDH1/2-mut WHO Grade 4). Landmark work demonstrated that IDH-mutant glioma cells acquire resistance to NK cell surveillance through epigenetic silencing of NKG2D ligands ULBP1 and ULBP3, driven by the 2-hydroxyglutarate (2-HG)-induced promoter hypermethylation that characterizes the G-CIMP methylator phenotype (50). Crucially, treatment with the hypomethylating agent decitabine (5-aza-2’-deoxycytidine) restores ULBP1/ULBP3 expression and sensitizes IDH-mutant glioma cells to NK cell-mediated lysis in an NKG2D-dependent manner (50). This finding establishes a direct mechanistic link between the oncometabolic driver of IDH-mutant glioma and NK cell evasion, and identifies epigenetic reprogramming as a viable strategy to restore innate immune surveillance.

In IDH wild-type GBM—the dominant subtype—NKG2D downregulation on NK cells is additionally mediated by a GBM-specific mechanism: tumor-derived lactate dehydrogenase 5 (LDH5) induces NKG2D ligand expression on GBM-associated monocytes, which in turn induce NKG2D receptor downregulation on NK cells through a trans-acting suppressive loop (51). This pathway illustrates the metabolic-epigenetic crosstalk unique to the GBM microenvironment that drives progressive NK cell dysfunction.

While single-cell transcriptomics has established the identities and relative proportions of cytotoxic lymphocyte subpopulations in GBM, it cannot reveal where these populations reside within the tumor—information that is critical for understanding both their functional state and their accessibility to therapy. Section 3 addresses this spatial dimension, examining how Tpex, Tex, and NK cell subsets are distributed across the perinecrotic, perivascular, and infiltrative-edge niches that define the tumor’s immunological geography.

### 3.3 Technical and interpretive caveats of single-cell and spatial multiomics in GBM

Although scRNA-seq and spatial transcriptomics have transformed the study of GBM immune ecology, their findings should be interpreted with caution. First, scRNA-seq is affected by dropout events, in which low-abundance transcripts may not be detected, and by batch effects arising from differences in tissue processing, dissociation protocols, sequencing depth, library preparation, and computational pipelines (32, 52–54). These limitations are particularly relevant for GBM cytotoxic lymphocyte studies because T cells and NK cells are often quantitatively scarce, and key transcripts such as cytokines, inhibitory receptors, transcription factors, and chemokines may be expressed at low levels. Therefore, absence of a transcript in a single-cell dataset should not be equated with biological absence of that molecule or cell state (32, 52–54).

Second, cell-state annotation is highly sensitive to analytical definitions. Tpex, Tint, Tex, TEMRA-like CD8^+^ T cells, and dysfunctional NK subsets are often defined by partially overlapping marker sets. For example, TCF7/TCF-1, PDCD1/PD-1, SLAMF6, CXCR5, CXCL13, GZMK, HAVCR2/TIM-3, LAG3, TIGIT, and GZMB may be weighted differently across studies. Consequently, cross-study comparison of Tpex: Tex ratios or NK dysfunction states requires caution unless datasets are harmonized using consistent preprocessing, batch correction, clustering, and annotation strategies (52–55).

Third, spatial transcriptomic platforms introduce additional constraints. Spot-based methods such as 10x Visium often capture RNA from multiple neighboring cells within a single spot, meaning that inferred cell–cell interactions or spatial niches may represent composite signals rather than direct single-cell contacts (56, 57). Spatial deconvolution can partially address this issue, but its accuracy depends on the quality of the single-cell reference atlas and may be limited when cell states are closely related, such as Tpex versus GZMK^+^ transitional CD8^+^ T cells or NK cells versus cytotoxic CD8^+^ T cells (57, 58). Thus, spatial niche assignments should be viewed as probabilistic and should ideally be validated by orthogonal protein-level approaches such as multiplexed immunofluorescence, CODEX, imaging mass cytometry, or spatial proteomics (59). scATAC-seq and multiome approaches remain underutilized in GBM immune studies but are essential for directly characterizing the epigenetic basis of Tpex versus Tex identity and for assessing the reversibility of exhaustion-associated chromatin states—a determinant of therapeutic responsiveness that transcriptional profiling alone cannot resolve.

Finally, most GBM single-cell and spatial studies remain limited by small cohort sizes, inter-patient variability, sampling bias, and incomplete clinical annotation. IDH status, MGMT(O^6^-methylguanine-DNA methyltransferase) promoter methylation, primary versus recurrent disease, prior radiotherapy or temozolomide exposure, corticosteroid use, surgical sampling region, and tissue ischemia time can all reshape the observed cytotoxic lymphocyte landscape. This concern is especially relevant in GBM, where anatomic transcriptional atlases, single-cell studies of the infiltrative front, and spatially resolved multiomics have shown substantial regional, cellular, and tumor–host heterogeneity (15, 22, 24, 25). Future studies should therefore report these variables systematically and avoid presenting cell-state proportions or spatial distributions as universal features of GBM unless validated across larger, treatment-stratified cohorts.

### 3.4 Temporal evolution of cytotoxic lymphocyte states in GBM

Most available single-cell and spatial studies of GBM provide cross-sectional snapshots, whereas cytotoxic lymphocyte states are likely to evolve during tumor progression, standard-of-care therapy, immune editing, and recurrence. Therefore, Tpex scarcity, Tex enrichment, GZMK^+^ transitional or effector-like expansion, and NK dysfunction should not be interpreted as fixed biological properties of GBM. These states may shift in response to antigen release, BBB/BTB disruption, myeloid recruitment, radiotherapy, temozolomide, corticosteroid exposure, and therapy-driven tumor-cell-state remodeling. Longitudinal single-cell studies support this dynamic view: in an immunocompetent EGFR-driven GBM model, immune composition changed during progression, with a transition from pro-inflammatory microglia to anti-inflammatory macrophages and protumorigenic myeloid-derived suppressor cells. Standard therapy also produced mixed immune effects, as temozolomide reduced MDSC accumulation, whereas combined temozolomide and irradiation increased intratumoral GranzymeB^+^ CD8^+^ T cells but also increased CD4^+^ regulatory T cells (60).

The temporal evolution of CD8^+^ T cells should be interpreted together with the developmental biology of exhaustion. During chronic antigen exposure, TCF-1^+^ Tex precursor cells can arise early and support downstream exhausted populations, whereas PD-1^+^ stem-like CD8^+^ T cells can generate proliferating transitory effector-like progeny before progression toward more terminal dysfunctional states (61, 62). However, this trajectory is not fully reversible. Tumor-specific CD8^+^ T cells may progress from a plastic dysfunctional state to a fixed dysfunctional state that resists reprogramming, and exhausted CD8^+^ T cells can acquire epigenetic stability that limits durable memory-like restoration after PD-1 pathway blockade (63, 64). This temporal constraint is particularly relevant at recurrence. Patient-matched primary–recurrent multiomic studies have shown that recurrent GBM often undergoes mesenchymal remodeling, and increased T-cell abundance at recurrence can be prognostic and associated with hypermutation status (65). Spatial analyses further suggest that recurrent tumors may develop remodeled perivascular niches and altered relationships among vascular features, immune infiltration, hypoxia, extracellular matrix architecture, and genetic alterations (66).

The temporal behavior of NK cells in GBM is less well defined but should also be considered dynamic. NK function may change across diagnosis, treatment, and recurrence through alterations in NKG2D ligand expression, HLA-E/NKG2A engagement, TIGIT/CD155 signaling, TGF-β exposure, lactate accumulation, corticosteroid use, and tumor-cell-state remodeling. At recurrence, therapy-driven antigen loss, vascular remodeling, myeloid expansion, and metabolic stress may further reduce NK-cell cytotoxic competence. This temporal framework has direct therapeutic implications: at diagnosis, strategies may aim to preserve residual Tpex-like cells and maintain NK activating-ligand recognition; during chemoradiation, immune monitoring should distinguish cytotoxic activation from expansion of Tregs, MDSCs, or suppressive macrophages; and at recurrence, spatial and clonal reassessment is essential. Future studies should incorporate paired diagnosis–recurrence sampling, matched blood and CSF profiling, TCR sequencing, single-cell and spatial multiomics, and treatment-timed pharmacodynamic sampling to determine whether cytotoxic lymphocytes are depleted, newly recruited, clonally expanded, reprogrammed, or spatially excluded over time (67).

### 3.5 Integrated summary: a context-dependent model of cytotoxic lymphocyte states in GBM

The single-cell atlas of GBM cytotoxic lymphocytes supports a context-dependent model rather than a fixed lineage map. CD8^+^ T cells and NK cells in GBM do not form uniform dysfunctional populations; instead, they occupy multiple partially overlapping states shaped by chronic antigen exposure, inhibitory receptor signaling, myeloid suppression, metabolic stress, tumor region, and treatment history. For CD8^+^ T cells, TCF7/TCF-1^+^PDCD1/PD-1^+^ Tpex-like cells may function as a progenitor-like reservoir, whereas GZMK^+^ transitional or effector-like cells, terminal Tex-like cells, and TEMRA-like cells represent downstream or parallel differentiation states whose proportions vary across cohorts. For NK cells, dysfunction is not equivalent to TCR-driven CD8^+^ T cell exhaustion, but reflects a distinct process involving inhibitory receptor dominance, activating ligand loss, metabolic suppression, maturation impairment, and tumor-induced reprogramming.

These cytotoxic lymphocyte states should be interpreted in relation to spatial and clinical context. Putative perivascular or APC-rich immune neighborhoods may support residual Tpex-like cells and local progenitor–progeny relationships, whereas perinecrotic and hypoxic regions may accelerate lymphocyte dysfunction through myeloid-derived suppression, IDO/kynurenine signaling, lactate accumulation, and TGF-β-driven metabolic stress. The infiltrative edge may represent a separate challenge, where partial BBB/BTB integrity, diffuse tumor invasion, and limited immune-cell access may contribute to spatial immune exclusion. Therefore, a single-cell annotation such as “Tpex,” “Tex,” “GZMK^+^ transitional,” or “stress-exhausted NK” should not be interpreted in isolation; it should be integrated with TCR clonality, spatial localization, tumor molecular subtype, IDH status, prior therapy, steroid exposure, and orthogonal protein-level validation.

This integrated model also provides a framework for therapeutic interpretation. Tpex-preserving strategies such as epigenetic priming, TCF-1/Wnt reinforcement, vaccination, or checkpoint modulation may be most relevant when a residual progenitor-like CD8^+^ T cell pool and supportive APC-rich niche are present. In contrast, tumors dominated by perinecrotic myeloid suppression, metabolic stress, or NK ligand loss may require myeloid reprogramming, metabolic intervention, restoration of activating NK ligands, or NK-directed checkpoint blockade before cytotoxic lymphocytes can be effectively mobilized. Thus, the therapeutic relevance of cytotoxic lymphocyte heterogeneity in GBM depends not only on the presence of specific immune cell states, but also on where these cells reside, which molecular programs constrain them, and whether the surrounding niche can support durable antitumor function.

Overall, Section 2 establishes that cytotoxic lymphocyte dysfunction in GBM is best viewed as a dynamic and spatially conditioned ecosystem. Single-cell and spatial multiomics provide essential tools for mapping this ecosystem, but their findings should be treated as hypothesis-generating unless validated across larger, clinically annotated, treatment-stratified, and spatially resolved cohorts. This framework sets the stage for the next section, where the anatomical organization of these cytotoxic lymphocyte states within perinecrotic, perivascular, and infiltrative-edge niches is considered in greater detail.

Critically, the dysfunctional states of CD8^+^ T cells and NK cells in GBM should not be interpreted as parallel but independent processes; they are embedded within—and mutually constrained by—a shared suppressive immune ecosystem. HMOX1^+^ TAMs simultaneously impair CD8^+^ T cell function through IL-10/STAT3 signaling and disable NK cell cytotoxicity by sustaining HLA-E expression that engages NKG2A on licensed NK cells. GBM-derived TGF-β1 jointly drives TOX-mediated epigenetic exhaustion in T cells and metabolic dysfunction with activating receptor downregulation in NK cells. The lactate- and PGE2-rich GBM microenvironment impairs CD8^+^ T cell glycolysis and NK cell calcium-dependent exocytosis through convergent but mechanistically distinct pathways. Spatially, perinecrotic HMOX1^+^ TAM-dominant zones exclude both terminally exhausted T cells and mature cytotoxic NK cells from productive tumor contact, while the perivascular niche preferentially maintains Tpex-like CD8^+^ T cells and may also harbor residual licensed NK subsets. The NK cell–cDC1 axis—whereby NK cells recruit conventional dendritic cells type 1 (cDC1) to the TME, thereby amplifying antigen cross-presentation and Tpex support—represents a further ecosystem-level connection that is disrupted in GBM (68). This ecosystem model implies that single-cell-type-directed therapies, even if mechanistically rational, are likely to fail if the surrounding suppressive architecture remains intact. Effective GBM immunotherapy must therefore simultaneously address the myeloid suppressive niche, NK cell licensing disruption, metabolic constraints, and T cell exhaustion programs—rather than targeting any single cytotoxic lymphocyte population in isolation.

While single-cell transcriptomics has established the identities and relative proportions of cytotoxic lymphocyte subpopulations in GBM, it cannot reveal where these populations reside within the tumor—information that is critical for understanding both their functional state and their accessibility to therapy. Section 3 addresses this spatial dimension, examining how Tpex, Tex, and NK cell subsets are distributed across the perinecrotic, perivascular, and infiltrative-edge niches that define the tumor’s immunological geography.

## 4 Spatial Contextualization of Cytotoxic Lymphocyte Dysfunction

### 4.1 Spatial transcriptomics technologies in GBM

**Figure 2:**
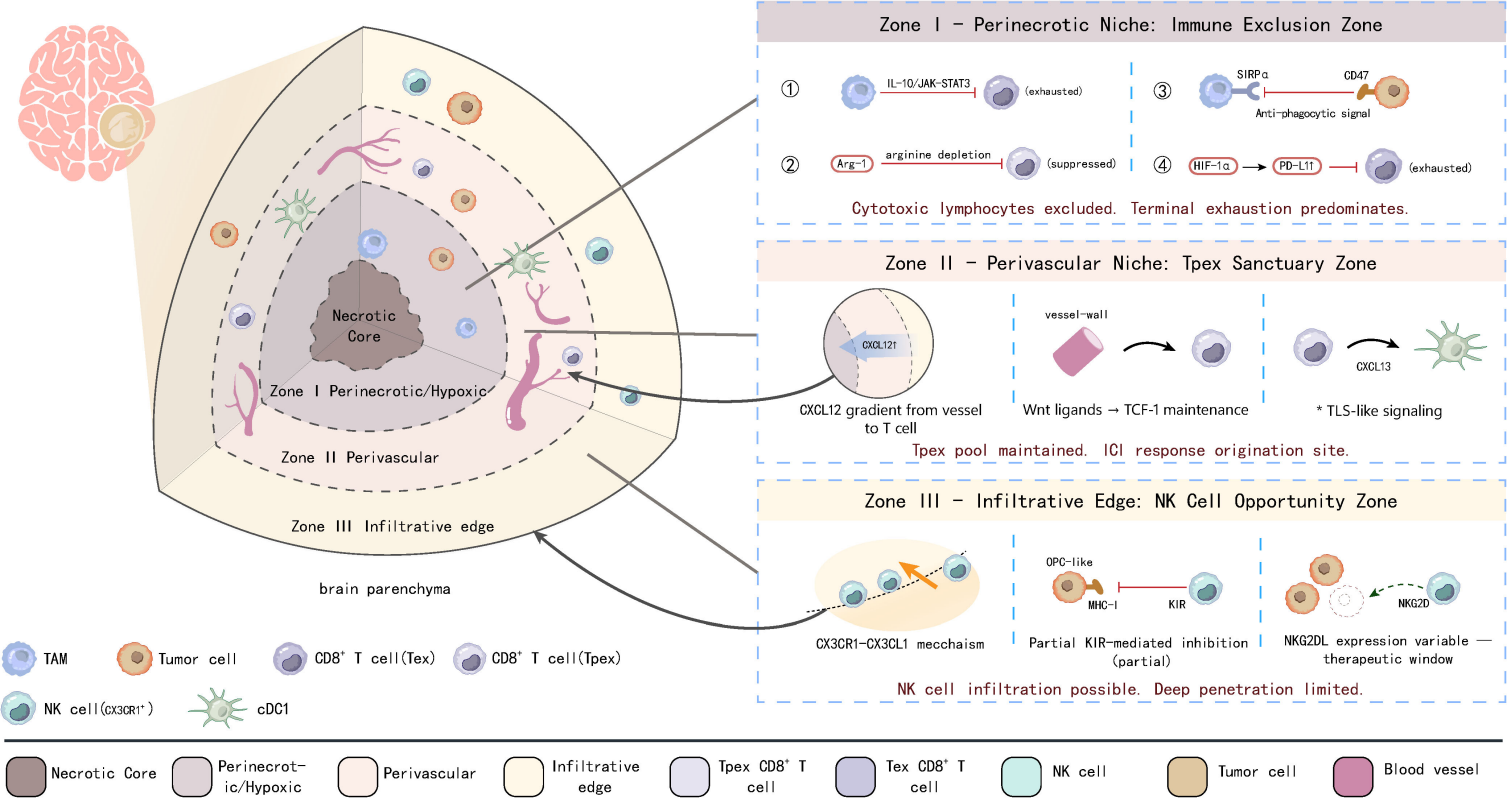
Spatial Immune Niche Architecture of the GBM Tumor Microenvironment. Spatial immune niche architecture of the glioblastoma tumor microenvironment and differential cytotoxic lymphocyte distribution. Cross-sectional schematic of a GBM tumor mass illustrating three spatially and functionally distinct immune niches. Zone I (perinecrotic/hypoxic, grey): predominant HMOX1^+^ immunosuppressive TAMs; near-complete exclusion of cytotoxic lymphocytes; residual CD8^+^ T cells undergo rapid terminal exhaustion driven by IL-10/JAK-STAT3, HIF-1α-mediated PD-L1 upregulation, and Arg-1. Zone II (perivascular, pale red): sanctuary for TCF-1^+^ Tpex cells, maintained through CXCL12 gradients, Wnt ligands, and CXCL13-cDC1 interactions; proposed as primary site for ICI response origination in GBM. Zone III (infiltrative edge, light yellow): relatively enriched for CX3CR1^+^ NK cells entering via CX3CR1/CX3CL1; deep penetration limited by MHC-I-mediated KIR inhibition and variable NKG2DL expression on OPC-like GBM cells. Callout boxes detail TAM-T cell suppressive crosstalk (IL-10, Arg-1, SIRPα-CD47); Tpex-cDC1-CXCL13 TLS-like signaling; CX3CR1/CX3CL1-driven NK cell entry at the infiltrative edge. TAM, tumor-associated macrophage; Tpex, progenitor-exhausted CD8^+^ T cell; cDC1, type 1 conventional dendritic cell; TLS, tertiary lymphoid structure; HIF-1α, hypoxia-inducible factor 1-alpha; HMOX1, heme oxygenase 1; OPC, oligodendrocyte precursor cell.

The application of spatially resolved transcriptomics and proteomics to GBM has created a new dimension of understanding of immune cell organization within the tumor (59). Technologies including 10x Genomics Visium (bulk-resolution spot-based), Xenium and MERFISH (single-cell resolution *in situ*), multiplexed immunofluorescence (CyCIF, CODEX), and mass spectrometry imaging (MALDI-MSI) have collectively revealed that GBM is organized into spatially distinct ecological niches that differ profoundly in cellular composition, transcriptional state, and immune permissiveness (7, 69). A landmark 2024 study integrating spatial transcriptomics and spatial proteomics across GBM patient cohorts identified three major modes of spatial organization—’cellular’, ‘hypoxic’, and ‘reactive’—each associated with distinct tumor cell states and immune infiltration patterns (8). Critically for cytotoxic lymphocyte biology, these niches were not uniformly distributed across tumor samples, and cytotoxic lymphocytes were largely excluded from the most hypoxic and necrotic regions. **Figure 2** demonstrates the spatial immune niche architecture of the GBM tumor microenvironment. According to the spatial heterogeneity of cellular landscape and behaviors patterns, the tumor tissue is stratified into three distinct microdomains.

### 4.2 The perinecrotic niche: a zone of cytotoxic exclusion

The perinecrotic niche—the region of pseudopalisading necrosis surrounded by hypoxic, viable tumor cells that is a histological hallmark of GBM—has been identified by spatial transcriptomics as a site of intense immunosuppression and near-complete exclusion of cytotoxic lymphocytes (17, 70). Analysis of paired snRNA-seq and Visium spatial transcriptomics data from GBM patients revealed that the perinecrotic niche is dominated by HMOX1^+^ immunosuppressive TAMs and hypoxia-responsive MES-like tumor cells, with CD8^+^ T cells and NK cells virtually absent from this zone despite their presence in adjacent viable tissue (17). Quantitatively, Visium-based spatial deconvolution from this cohort showed that perinecrotic spots contained less than 5% cytotoxic lymphocyte signal versus 25–35% immunosuppressive macrophage signal, with a TAM: CD8^+^ T cell spatial enrichment ratio exceeding 6:1 within pseudopalisading regions (17). The spatial segregation of cytotoxic lymphocytes away from the necrotic core likely reflects a combination of factors: hypoxia-driven HIF-1α upregulation of PD-L1 on tumor and myeloid cells; Arg-1-mediated arginine depletion that starves T cells of a critical amino acid; and TGF-β gradients emanating from pseudopalisading cells that enforce T cell motility arrest.

This spatial exclusion has direct neurosurgical relevance: the perinecrotic niche corresponds to the most treatment-resistant region of GBM, and its immune exclusion suggests that systemic ICI alone will be insufficient to engage anti-tumor immunity in this zone. Strategies to repolarize the perinecrotic TAM niche—or to engineer cytotoxic lymphocytes capable of tolerating its hypoxic and metabolic hostility—represent a critical unmet need.

### 4.3 The perivascular niche: a refuge for progenitor-exhausted T cells

In striking contrast to the necrotic core, the perivascular niche—the area surrounding intratumoral blood vessels—emerges as a relative sanctuary for TCF-1^+^ progenitor-exhausted CD8^+^ T cells (Tpex) in GBM (66). Spatially resolved profiling of GBM tissues has confirmed that the perivascular space is enriched for immune cells in general, and spatial immune profiling has identified an ‘inflammatory perivascular phenotype’ associated with significantly longer patient survival: median overall survival of 22.9 months versus 12.3 months in patients lacking this phenotype (HR = 0.44, 95% CI 0.22–0.87) (71). The perivascular niche provides Tpex-supporting cues including proximity to vasculature-derived CXCL12 and Wnt ligands that maintain TCF-1 expression, as well as access to antigen-presenting cDC1 cells that prevent premature Tpex-to-Tex transition (72). These observations suggest that the perivascular niche functions as a de facto ‘tertiary lymphoid structure surrogate’ in GBM—the location at which residual immune competence is maintained and from which immunotherapy responses, if they occur, must originate.

The spatial mapping of Tpex cells to the perivascular niche also has therapeutic implications for surgery: maximal safe resection that necessarily sacrifices perivascular tissue may inadvertently deplete the residual Tpex pool, potentially compromising the capacity for post-operative immunotherapy response. This hypothesis warrants prospective investigation through longitudinal paired profiling of pre- and post-operative GBM specimens.

### 4.4 The infiltrative edge: NK cell opportunity zone

The tumor-brain interface—the infiltrative edge where GBM cells diffusely invade non-neoplastic brain—represents a third distinct immune niche with unique features relevant to cytotoxic lymphocyte biology. Spatial analyses indicate that the infiltrative edge is relatively enriched for CX3CR1^+^ NK cells compared to the tumor core, consistent with the preference of these cells for perivascular and parenchymal entry routes (13, 73). The lower density of immunosuppressive TAMs and the relatively preserved BBB-associated vasculature in this zone may create a less suppressive microenvironment for NK cell function, at least transiently. However, the infiltrative edge also features OPC-like and neuron-interacting GBM cells with high MHC-I expression and low NKG2D ligand expression, which may render them relatively resistant to NK cell cytotoxicity through the ‘missing self’ mechanism operating in reverse.

The spatial enrichment of NK cells at the infiltrative edge, combined with their relative depletion from the tumor core, suggests a model in which NK cells are capable of entering the CNS but fail to penetrate deeply into the immunosuppressive tumor mass. Strategies to enhance NK cell persistence and deep tumor penetration—such as anti-TIGIT therapy combined with CCL5-engineered NK cells—may be particularly effective if directed at the infiltrative edge.

### 4.5 Intercellular crosstalk: TAM–CTL spatial interactions

Spatial cell-cell interaction analysis has identified several key communication axes between TAMs and cytotoxic lymphocytes in GBM that drive immune suppression (74). The HMOX1^+^ TAM → exhausted CD8^+^ T cell axis, mediated by IL-10/JAK-STAT3 signaling, represents a spatially dominant suppressive interaction in the perinecrotic niche (17). Additionally, the SIRPα (TAM)–CD47 (GBM/immune cells) axis suppresses both phagocytic activity of TAMs and, through competitive CD47 expression on cytotoxic lymphocytes, reduces their survival in the tumor bed (75). The galectin-1–CD8 axis—whereby GBM-derived galectin-1 binds to T cell surface glycoproteins and induces apoptosis—has been spatially mapped to the MES-like tumor region (76). Together, these data paint a picture of a spatially organized suppressive network in which specific intercellular interactions are concentrated in defined tumor regions, opening the possibility of spatially targeted combination therapies.

Spatial mapping thus delineates the geography of cytotoxic immune failure in GBM. However, understanding why these dysfunctional states are sustained—and in many cases, irreversible—requires examination of the intracellular molecular machinery that underlies exhaustion. The following section addresses the epigenetic and transcriptional reprogramming events within CD8^+^ T cells and NK cells that constitute the durable molecular basis of cytotoxic failure in GBM.

## 5 Epigenetic and Transcriptional Reprogramming in GBM: CNS-Specific Dimensions

### 5.1 The TOX–TCF-1 epigenetic axis in GBM CD8^+^ T cells

Epigenetic reprogramming is the molecular cornerstone of T cell exhaustion, distinguishing it from mere functional anergy by creating a heritable, largely irreversible transcriptional state. In GBM, the TOX transcription factor—induced by chronic TCR stimulation in combination with co-inhibitory receptor signaling—drives the epigenetic ‘locking’ of the exhausted state through: (i) induction of H3K27me3 marks at effector gene loci (PRF1, IFNG, TNF, GZMB); (ii) deposition of the exhaustion-associated chromatin signature at PDCD1 (PD-1) and HAVCR2 (TIM-3) regulatory elements; and (iii) suppression of TCF-1 (TCF7) expression, thereby blocking the Tpex self-renewal program (77, 78).

Single-cell transcriptome analysis across multiple cancer types confirmed that TOX expression level is positively correlated with exhaustion severity as defined by PD-1 expression, and that TOX knockdown in tumor-infiltrating CD8^+^ T cells significantly reduces expression of PD-1, TIM-3, TIGIT, and CTLA-4 (79). In GBM specifically, the TOX-driven exhaustion program is amplified by several CNS-specific factors: (i) TGF-β-driven TOX induction through SMAD2/3 → IRF4 → TOX cascade; (ii) hypoxia-mediated HIF-1α synergy with TOX-induced epigenetic changes; and (iii) the near-absence of IL-21 (which normally counteracts TOX activity in follicular-rich environments) due to the scarcity of intratumoral TFH cells in the GBM TME (80).

NR4A transcription factors (NR4A1, NR4A2, NR4A3) cooperate with TOX to enforce terminal exhaustion by repressing NFAT-driven effector programs. While NR4A expression does not consistently correlate with exhaustion severity in isolation, combined TOX/NR4A co-expression represents the fully committed terminal exhausted state that is epigenetically irreversible with current checkpoint blockade approaches (78). These data identify TOX/NR4A as rational targets for epigenetic intervention strategies in GBM, as discussed in Section 6.

### 5.2 Epigenetic regulation of NK cell ligands in GBM

NK cell dysfunction in GBM is substantially mediated by epigenetic silencing of activating ligands on tumor cells, rather than solely by NK cell-intrinsic reprogramming. The NKG2D ligand family—including MICA, MICB, and the UL16-binding proteins ULBP1–6—are normally expressed on stressed and transformed cells and trigger cytotoxicity by NK cells and CD8^+^ T cells via the NKG2D receptor. In GBM, expression of these ligands is suppressed through three overlapping mechanisms: (1) promoter hypermethylation, particularly of ULBP1 and ULBP3 in IDH-mutant gliomas, driven by 2-hydroxyglutarate (2-HG)-mediated DNA methyltransferase (DNMT) activity; (2) histone deacetylation of NKG2D ligand gene loci by histone deacetylases (HDACs) enriched in the GBM tumor microenvironment; and (3) microRNA-mediated post-transcriptional silencing (50, 70, 81).

On the NK cell side, epigenetic reprogramming in the GBM TME includes: lactate-driven HDAC inhibition that paradoxically promotes NKG2D receptor promoter methylation—a form of metabolic-epigenetic crosstalk; TGF-β1-induced chromatin remodeling that suppresses DNAM-1 (CD226) expression; and hypoxia-mediated HIF-1α activation that upregulates inhibitory KIR expression and downregulates NKp30/NKp46 activating receptor surface density (14, 45, 82). The convergence of tumor-side ligand silencing and NK cell-side receptor epigenetic dysregulation creates a dual blockade of NK cell activation signals that is particularly difficult to overcome with single-agent immunotherapy.

### 5.3 Mechanosensory epigenetic regulation: a CNS-specific dimension

A recently identified CNS-specific epigenetic mechanism involves the mechanosensory ion channel Piezo1 and its downstream transcriptional target Osr2. Elevated interstitial pressure—a near-universal feature of high-grade GBM due to the tumor’s growth within the fixed cranial vault—activates Piezo1 in tumor-infiltrating T cells, triggering a calcium influx that activates calcineurin/NFAT signaling and promotes premature terminal differentiation. The Osr2 transcription factor, downstream of Piezo1, directly represses TCF7 (TCF-1) expression and accelerates the Tpex-to-Tex transition (20). This pathway, largely absent or less relevant in soft-tissue tumors with lower interstitial pressure, represents a truly CNS- and GBM-specific mechanism of cytotoxic T cell failure that warrants targeted investigation and potential pharmacological intervention via Piezo1 antagonists in the neurosurgical context.

Together, the epigenetic and transcriptional programs reviewed in this section—TOX/NR4A-driven T cell exhaustion, tumor-side NKG2D ligand silencing, and Piezo1-mediated mechanosensory Tpex depletion—provide a mechanistic explanation for why immune checkpoint blockade has achieved limited efficacy in GBM. They also define molecular leverage points that may be targeted by rational, multimodal immunotherapy strategies. Section 5 examines the clinical evidence for ICI failure and outlines how insights from single-cell, spatial, and epigenetic biology can be translated into neurosurgically relevant therapeutic approaches.

## 6 Translational implications and neurosurgically relevant therapeutic strategies

### 6.1 Clinical evidence: why immune checkpoint blockade has failed in unselected GBM

Despite strong biological rationale, immune checkpoint blockade has not improved survival in immune-biomarker-unselected GBM populations. In the randomized phase III CheckMate-143 trial, nivolumab failed to improve overall survival compared with bevacizumab in recurrent GBM; median overall survival was 9.8 months with nivolumab and 10.0 months with bevacizumab, and objective response rate was lower in the nivolumab arm (83). Negative results were also observed in newly diagnosed GBM. In CheckMate-498, nivolumab plus radiotherapy did not improve survival compared with temozolomide plus radiotherapy in MGMT-unmethylated GBM (84).In CheckMate-548, adding nivolumab to radiotherapy plus temozolomide in newly diagnosed MGMT-methylated or indeterminate GBM did not improve progression-free or overall survival and increased grade 3–4 treatment-related adverse events (85). Collectively, these trials show that PD-1 blockade, whether used at recurrence or combined with standard radiotherapy or chemoradiotherapy, has not been effective in GBM populations not selected by cytotoxic lymphocyte biomarkers or spatial immune niche features.

The failure of ICIs in GBM should not be interpreted as absence of PD-1 biology, but rather as evidence that checkpoint blockade alone cannot overcome the unique immunological architecture of this tumor (86). Compared with ICI-responsive tumors such as melanoma and NSCLC, GBM is characterized by lower cytotoxic lymphocyte abundance, myeloid-dominant immunosuppression, limited Tpex-supportive or TLS-like niches, BBB/BTB constraints, frequent corticosteroid exposure, spatial immune exclusion, and potentially insufficient Tpex reservoirs for checkpoint-driven proliferative reinvigoration (67, 86). In melanoma, TCF7^+^ memory-like CD8^+^ T cell states and a higher TCF7^+^CD8^+^/TCF7^-^CD8^+^ ratio are associated with anti-PD-1 response and improved survival (39). In NSCLC, a transcriptionally and functionally distinct tumor-reactive PD-1^+^CD8^+^ T cell pool has also been associated with response and survival after PD-1 blockade (87). In GBM, however, this cellular substrate may be quantitatively constrained, spatially inaccessible, metabolically compromised, or disconnected from APC-rich niches that maintain TCF1^+^ stem-like CD8^+^ T cells in other human tumors (38).

Importantly, Tpex/Tex balance has not yet been prospectively incorporated as a biomarker in major GBM checkpoint blockade trials. Therefore, the relationship between Tpex abundance, Tpex: Tex ratio, TCR clonality, APC-rich niche support, and clinical response remains a mechanistically grounded but clinically unvalidated hypothesis in GBM. Future checkpoint trials should stratify patients by cytotoxic lymphocyte states, steroid exposure, TCR clonality, myeloid suppressive burden, and spatial immune exclusion, rather than relying only on histological diagnosis, recurrence status, or MGMT promoter methylation. Such stratification may help distinguish true PD-1 resistance from the absence of a progenitor-like CD8^+^ T cell substrate required for checkpoint-driven reinvigoration.

NK cell-based clinical translation is also early but informative. In a first-in-human phase I study of recurrent HER2-positive GBM, intracranial injection of HER2-targeted CAR-NK cells during relapse surgery was feasible and safe, with no dose-limiting toxicities, cytokine release syndrome, or immune effector cell-associated neurotoxicity syndrome. However, durable efficacy was limited: five patients achieved stable disease lasting 7–37 weeks, four had progressive disease, median progression-free survival was 7 weeks, and median overall survival was 31 weeks. Notably, pre-treatment CD8^+^ T cell infiltration in recurrent tumor tissue positively correlated with time to progression (Spearman r = 0.71, p < 0.05), and among the nine enrolled patients, five achieved stable disease (median duration 18 weeks) while four had progressive disease, with a median progression-free survival of 7 weeks and median overall survival of 31 weeks—suggesting that NK-based cellular therapy may still depend on the broader cytotoxic immune ecosystem rather than NK activity alone (88). These observations support a spatial interpretation of therapy resistance: systemic ICI or cellular therapy may fail despite the presence of biologically relevant immune targets if cytotoxic lymphocytes are scarce, spatially excluded, metabolically compromised, or disconnected from supportive APC-rich niches.

These clinical failures should also temper the therapeutic strategies proposed below. Epigenetic priming agents—including decitabine, LSD1 inhibitors, and BET bromodomain inhibitors—do not act selectively on tumor-infiltrating immune cells. They can simultaneously affect malignant cells, myeloid cells, endothelial cells, lymphocytes, and normal proliferating tissues. Their activity should therefore not be assumed to specifically restore GBM-infiltrating Tpex cells or NK-cell function. TCF-1/Wnt-based Tpex-restorative strategies also require caution: broad systemic Wnt/β-catenin activation may promote tumor-intrinsic immune exclusion. Safer approaches would be T cell-restricted, transient, locally controlled, or integrated into ex vivo cellular engineering workflows (89). Combinatorial cell-cycle blockade likewise remains uncertain in GBM. Although CDK4/6 inhibition can enhance tumor antigen presentation and antitumor immunity in preclinical models, palbociclib monotherapy was ineffective in recurrent RB1-positive GBM despite molecular selection, underscoring the need to consider RB-pathway integrity, CNS drug exposure, hematologic toxicity, and effects on proliferating immune cells (90).

The biology of the blood-brain barrier (BBB) and blood-tumor barrier (BTB) remains a major translational constraint for GBM immunotherapy. Although the contrast-enhancing tumor core may exhibit partial BTB disruption, the infiltrative edge and peritumoral brain often retain more intact barrier properties. Consequently, systemically administered agents—including antibodies, epigenetic drugs, cytokines, nanoparticles, kinase inhibitors, and engineered immune cells—may distribute unevenly across GBM regions. Local or regional delivery strategies may partially address this limitation. These include resection cavity-based biomaterials, intracavitary or intraventricular cellular therapy, Ommaya reservoir-based administration, convection-enhanced delivery, and perioperative drug release. However, such approaches are not a complete solution to GBM immune resistance. They introduce their own practical challenges: heterogeneous drug distribution, catheter or cavity geometry constraints, local edema or inflammation, pseudoprogression, difficulties with repeat dosing, and uncertainty in pharmacodynamic target engagement. Future GBM immunotherapy trials should therefore incorporate tissue pharmacodynamics, paired blood-tumor-CSF immune monitoring, spatial validation of target engagement, and region-specific pharmacodynamic endpoints, rather than relying solely on systemic dosing and radiographic response (91, 92).

### 6.2 Targeting the Tpex pool: epigenetic priming and checkpoint combination

**Figure 3:**
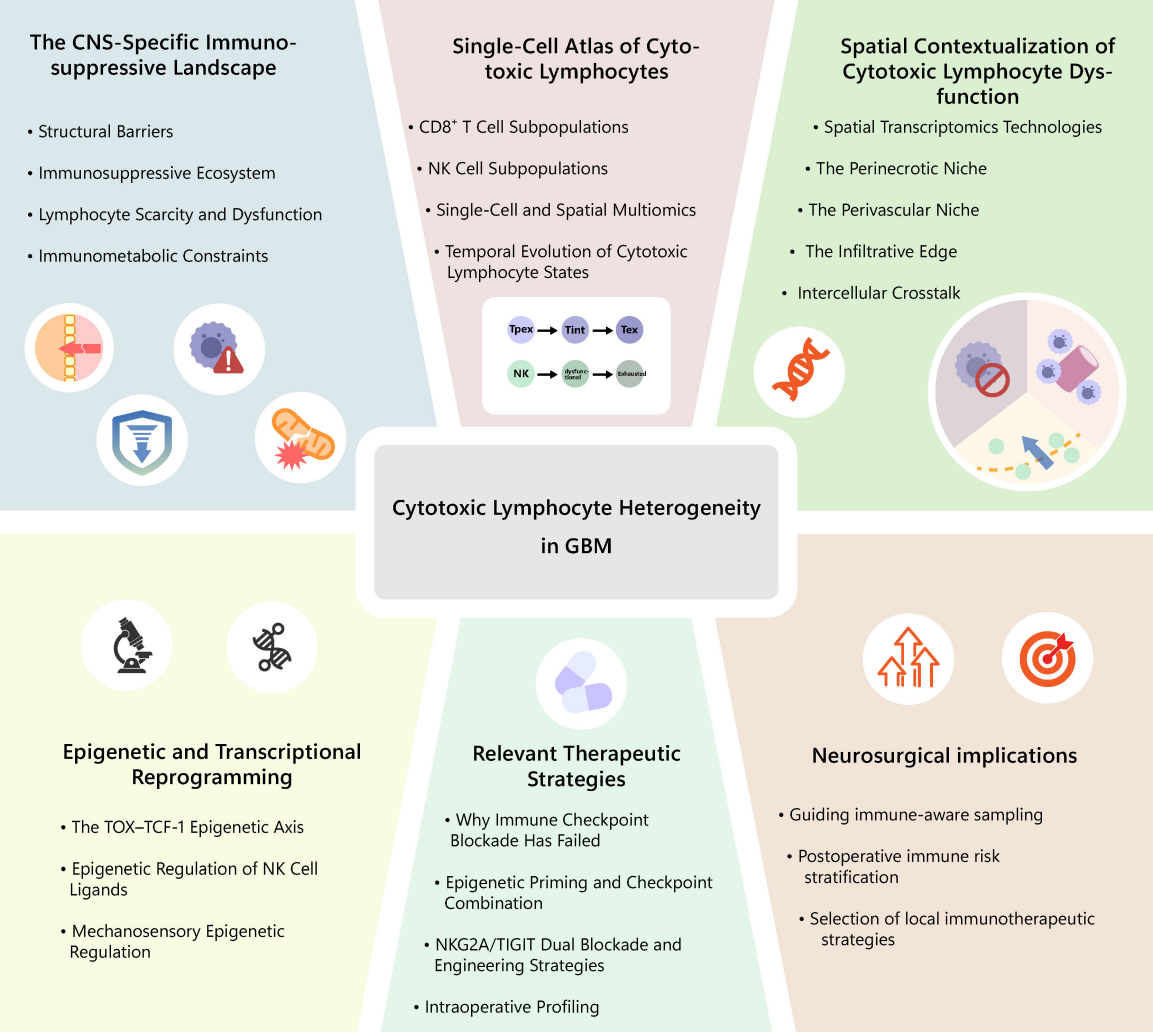
Integrated Multiomics-to-Therapy Framework for Cytotoxic Lymphocyte Reprogramming in GBM. Integrated multiomics-guided therapeutic reprogramming framework for cytotoxic lymphocyte reinvigoration in glioblastoma. A four-stage translational pipeline linking intraoperative spatial multiomics profiling to neurosurgically tailored immunotherapy delivery. Stage 1 (Profiling): GBM specimens subjected to scRNA-seq, spatially resolved transcriptomics (Visium/Xenium), and epigenomic profiling (ATAC-seq) to generate comprehensive immune landscape including Tpex:Tex ratio, NK infiltration density, IDH status, and niche organization. Stage 2 (Stratification): Patient-specific therapeutic targets identified in CD8^+^ T cells (TOX/NR4A locking, Tpex depletion, Piezo1-TGF-β) and NK cells (NKG2A/HLA-E and TIGIT/CD155 axes, NKG2DL silencing, mitochondrial dysfunction). Stage 3 (Reprogramming): For CD8^+^: low-dose decitabine + LSD1 inhibition + anti-PD-1/anti-TIM-3 checkpoint blockade. For NK: anti-NKG2A (monalizumab) + anti-EGFR (cetuximab; ADCC); anti-TIGIT/PD-1/CD73; CRISPR iPSC-CAR-NK (ΔNKG2A ΔTIGIT). Decitabine priming proposed to enhance Tpex expansion and CAR-NK efficacy synergistically. Stage 4 (Delivery): Post-resection intracavitary delivery via Ommaya reservoir bypasses the BBB; systemic ICI initiated adjuvantly. Timeline: pre-operative profiling → surgical resection → intracavitary Day 1–3 → systemic ICI Day 14+. ICI, immune checkpoint inhibitor; ADCC, antibody-dependent cellular cytotoxicity; CAR-NK, chimeric antigen receptor NK; iPSC, induced pluripotent stem cell; LSD1, lysine-specific demethylase 1; BBB, blood-brain barrier.

Given the critical dependence of ICI responses on a residual Tpex pool, strategies to expand or preserve Tpex in GBM are of high translational priority. Decitabine (5-aza-2’-deoxycytidine), a DNA methyltransferase inhibitor, has shown dual utility in this context: at low, ‘epigenetic priming’ doses, it reduces PDCD1 promoter methylation (increasing PD-1 expression paradoxically, while simultaneously reducing terminal exhaustion epigenetic marks), expands the Tpex fraction, and enhances anti-PD-1 efficacy in preclinical solid tumor models (43). In the specific context of IDH-mutant glioma/GBM, decitabine additionally reverses NKG2D ligand silencing and restores NK cell immunosurveillance, offering dual benefit (50). Clinical studies combining decitabine with ICI in GBM are therefore biologically rational, but should be designed with paired immune monitoring to determine whether Tpex-directed effects occur systemically, locally, or both. **Figure 3** demonstrates a translational therapeutic framework based on integrated multiomics for GBM cytotoxic lymphocyte reprogramming, leveraging the dual therapeutic benefits of decitabine.

LSD1 (KDM1A) inhibition represents another epigenetic approach to Tpex preservation: LSD1 inhibitors reduce H3K4me1 marks at TCF7 regulatory elements, maintain TCF-1 expression, sustain the Tpex fraction, and produce durable responses to PD-1 blockade in preclinical models through Tpex pool preservation rather than direct T cell reinvigoration (93). BET bromodomain inhibitors similarly enhance TCF7 accessibility in exhausted T cells and have shown efficacy in enhancing anti-PD-1 responses in preclinical models (94). The combination of these epigenetic agents with standard GBM chemoradiation warrants careful investigation, particularly in the neoadjuvant or perioperative setting where tumor-specific immune responses may be most readily mobilized (95).

An important unresolved issue is whether decitabine or LSD1 inhibition acts on Tpex-like cells in GBM through peripheral expansion, local intratumoral maintenance, or both. Current evidence does not yet demonstrate that either agent directly expands brain-resident or GBM-infiltrating Tpex cells in human GBM. Rather, available evidence supports the broader concept that epigenetic priming can preserve or expand progenitor-exhausted CD8^+^ T cell states in tumor models. In GBM, two non-mutually exclusive mechanisms should therefore be considered. First, systemic epigenetic priming may preserve or expand tumor-reactive Tpex cells in peripheral immune compartments, including blood, bone marrow, spleen, lymph nodes, or cervical draining lymph nodes, followed by trafficking into the CNS tumor through permissive vascular or perivascular routes. Second, if sufficient drug exposure is achieved across the disrupted blood–tumor barrier or within the postoperative tumor bed, epigenetic priming may locally maintain TCF7/TCF-1-associated progenitor programs in GBM-infiltrating Tpex-like cells, particularly within perivascular or APC-rich immune niches.

The local mechanism is biologically plausible but remains unproven in GBM. Intratumoral Tcf1^+^PD-1^+^CD8^+^ T cells have been shown in tumor models to possess expansion, regeneration, and differentiation capacity and to mediate tumor control after vaccination or checkpoint blockade (37). Human tumor studies further indicate that TCF1^+^ stem-like CD8^+^ T cells can be maintained in dense MHC-II^+^ antigen-presenting-cell niches and clonally generate more terminally differentiated checkpoint-high progeny (38). However, whether analogous Tpex-supportive niches exist in GBM, and whether they are pharmacologically expanded or stabilized by decitabine or LSD1 inhibition, remains to be determined. This distinction is particularly important in GBM because BBB/BTB permeability, corticosteroid exposure, local drug penetration, tumor vascularity, and the spatial availability of residual Tpex-supportive niches may strongly influence therapeutic effects.

Future GBM studies should explicitly distinguish these mechanisms by integrating paired blood–tumor–CSF sampling, single-cell RNA/TCR sequencing, spatial transcriptomics or spatial proteomics, pharmacodynamic assessment of drug exposure within GBM tissue, and longitudinal immune profiling before and after epigenetic priming. Tracking shared TCR clonotypes between peripheral Tpex-like cells and GBM-infiltrating T cells would be particularly informative for determining whether treatment-expanded Tpex cells originate outside the CNS and subsequently traffic into the tumor, or whether they are locally maintained and expanded within the GBM microenvironment. Until such data are available, decitabine and LSD1 inhibition should be presented as promising Tpex-preserving strategies whose GBM-specific anatomical site of action remains unresolved, rather than as established methods for directly restoring brain-resident Tpex cells.

In addition to epigenetic priming, T cell-directed reinforcement of the TCF7/TCF-1–Wnt axis represents a potential Tpex-restorative strategy for GBM. Candidate molecular nodes include TCF7/TCF-1 itself, LEF1, WNT3A–FZD–LRP5/6 signaling, β-catenin stabilization through GSK3β inhibition or AXIN–β-catenin modulation, and Wnt-associated epigenetic regulators such as PRMT1. Downstream TCF-1-dependent persistence programs, including EOMES-associated Tex precursor maintenance and the c-Myb–BCL2 survival axis, may also help sustain a progenitor-like exhausted CD8^+^ T cell pool. The goal of this strategy is not to claim definitive conversion of epigenetically fixed terminal Tex cells into bona fide Tpex cells *in vivo*, but rather to preserve residual Tpex cells, reinforce progenitor-like programs in less differentiated exhausted or GZMK^+^ transitional CD8^+^ T cells, prevent premature Tpex-to-Tex transition, restore APC-rich niches that support stem-like CD8^+^ T cells, or generate Tpex-like CAR-T/TCR-engineered T cells ex vivo before adoptive transfer. This rationale is supported by studies showing that intratumoral Tcf1^+^PD-1^+^CD8^+^ T cells possess expansion, regeneration, and differentiation capacity; that TCF1^+^ stem-like CD8^+^ T cells can be maintained within APC-rich intratumoral niches; and that TCF-1 is central to early exhausted CD8^+^ T cell fate decisions. Wnt/β-catenin activation can restrain effector differentiation and promote CD8^+^ memory stem-like programs, while ectopic Tcf1 expression can instill stem-like properties in exhausted CD8^+^ T cells in chronic infection and preclinical tumor models (37, 38, 61, 63, 88, 96–98).

An important translational constraint is that TCF-1/Wnt-based strategies should preferentially target residual Tpex-like or transitional exhausted cells rather than attempt reversal of terminally exhausted Tex cells, given the fixed epigenetic states that limit durable reprogramming of the latter. Broad systemic Wnt/β-catenin activation should also be avoided, as tumor-intrinsic β-catenin signaling can promote T cell exclusion. Safer approaches for GBM include T cell-restricted TCF7/TCF-1 reinforcement, transient ex vivo Wnt modulation during CAR-T manufacturing, and locally controlled or inducible delivery platforms. In **Table 1**, this strategy is therefore listed as “T cell-directed TCF-1/Wnt reinforcement or Tpex-restorative programming.” (63, 88, 98).

**Table 1:** Summary of therapeutic strategies.

Strategy	Target	Mechanism	GBM-Specific rationale
Decitabine + anti-PD-1	Tpex pool expansion + checkpoint	DNMT inhibition restores TCF-1, reverses NKG2D ligand silencing	Low Tpex in GBM; IDH-mut NK evasion
LSD1 inhibitor + ICI	Tpex epigenetic preservation	Maintains H3K4me1 at TCF7, sustains Tpex fraction	Accelerated Tpex→Tex transition in CNS
Anti-NKG2A (monalizumab) + anti-EGFR	NK cell NKG2A/HLA-E axis	Relieves dominant NK inhibitory signal; ADCC via cetuximab	EGFR amplification in ~40% GBM; HLA-E upregulation
Anti-TIGIT + anti-PD-1 + anti-CD73	T cell + NK checkpoint + adenosine	Dual effector reinvigoration + metabolic reprogramming	TIGIT co-expressed on GBM-infiltrating T and NK cells
CRISPR-engineered iPSC-CAR-NK	GBM antigens (EGFRvIII, IL13Rα2)	Multi-checkpoint deletion + antigen targeting	Overcomes NK cell-intrinsic exhaustion programs
Intracavitary NK/T cell delivery	Tumor resection cavity	Local high-concentration delivery post-surgery	BBB bypass; surgical opportunity window
Intraoperative immune profiling	Tpex: Tex ratio, NK density, IDH status	Personalized immunotherapy selection	Neurosurgical access to fresh tumor tissue

### 6.3 NK cell reactivation: NKG2A/TIGIT dual blockade and engineering strategies

NK cell-directed immunotherapy in GBM is an emerging field with several promising avenues. The NKG2A/HLA-E inhibitory axis—which dominantly suppresses NK cells in multiple tumor types through engagement of the CD94-NKG2A heterodimer on NK cells with HLA-E overexpressed on tumor cells—is a therapeutically actionable target in GBM (47). Monalizumab, an anti-NKG2A antibody, has demonstrated preclinical activity in combination with cetuximab (anti-EGFR) in EGFR-amplified GBM—one of the most common molecular alterations in GBM—by simultaneously blocking the HLA-E inhibitory signal on NK cells and promoting antibody-dependent cellular cytotoxicity (ADCC) through EGFR targeting (99).

TIGIT blockade represents a complementary approach, as TIGIT is co-expressed with PD-1 on both exhausted CD8^+^ T cells and dysfunctional NK cells in GBM, and its ligand CD155 is highly expressed on GBM tumor cells and TAMs (19, 100). Dual anti-PD-1/anti-TIGIT therapy significantly improved survival and enhanced effector T cell function while reducing suppressive Tregs in preclinical GBM models, and was further potentiated by co-targeting CD73 to reverse adenosine-mediated immunosuppression. The translational development of NKG2A + TIGIT dual blockade—targeting both the dominant NK cell and T cell inhibitory axes—represents a rationally designed GBM-specific combination strategy.

CAR-NK cell engineering offers additional opportunities tailored to the GBM TME. iPSC-derived NK cells engineered with synNotch-controlled CAR constructs targeting GBM antigens (EGFRvIII, IL13Rα2, GD2) and equipped with CRISPR-deleted NKG2A and TIGIT genes—removing cell-intrinsic brakes—represent a fully engineered approach to overcome the multiple layers of NK cell suppression in GBM (101). The neurosurgical operative field provides a unique and clinically accessible route for local delivery: direct intracavitary injection into the tumor resection cavity, potentially via Ommaya reservoir, could achieve high local concentrations of engineered NK cells while minimizing systemic toxicity.

### 6.4 Spatial multiomics-guided intraoperative profiling

Perhaps the most direct translational application of the spatial multiomics insights reviewed here lies in real-time intraoperative tumor profiling to guide personalized immunotherapy decisions. The neurosurgical resection of GBM provides a time-limited but clinically precious opportunity to characterize the immune landscape of the tumor—including the Tpex: Tex ratio, NK cell infiltration density, spatial distribution of immune niches, and IDH mutation status—in a manner that can directly inform adjuvant immunotherapy selection (102). Emerging rapid scRNA-seq workflows capable of generating single-cell transcriptomic data within 6–8 hours of surgical specimen acquisition, combined with intraoperative mass spectrometry imaging for metabolic profiling, could realistically enable same-procedure immune profiling and immunotherapy stratification in the near future (103).

Furthermore, longitudinal tissue sampling at primary surgery and recurrence—a practice already incorporated in some GBM clinical trial protocols—combined with paired scRNA-seq and spatial transcriptomics, would enable dynamic tracking of cytotoxic lymphocyte subpopulation evolution in response to treatment. Such longitudinal multiomics datasets would be invaluable for understanding immunotherapy resistance mechanisms in GBM and identifying early biomarkers of response or failure.

Spatial multiomics data further offer a framework for spatially stratified immunotherapy delivery. Rather than applying systemic immunotherapy uniformly, spatial immune niche mapping may guide region-specific intervention: perivascular Tpex-enriched zones may benefit from local checkpoint modulation or epigenetic priming to preserve the progenitor pool; perinecrotic myeloid-dominant zones may require myeloid reprogramming agents (e.g., CSF1R blockade, anti-CD47) or metabolic interventions prior to cytotoxic lymphocyte-directed therapy; and the infiltrative edge may be the most accessible target for NK cell-directed strategies. Clinically feasible delivery platforms—including resection cavity biomaterials, Ommaya reservoir administration, and convection-enhanced delivery—can be spatially informed by preoperative MRI-radiomics and intraoperative immune profiling to match treatment to the dominant suppressive architecture of each patient’s tumor.

Many of the translational strategies outlined in this section are uniquely enabled—or constrained—by the neurosurgical context of GBM management. The operative resection of GBM provides a time-limited but clinically invaluable window for spatial immune profiling, local therapeutic delivery, and perioperative immune monitoring that has no direct parallel in other solid tumor indications. Section 6 addresses how spatial cytotoxic lymphocyte mapping can be integrated into neurosurgical practice to guide sampling strategy, postoperative immunotherapy selection, and intracavitary delivery of precision immunotherapeutic agents.

## 7 Neurosurgical implications of spatial cytotoxic lymphocyte mapping

Spatial cytotoxic lymphocyte mapping has practical neurosurgical relevance, but it should not be interpreted as a rationale to reduce the extent of oncologically indicated resection. Maximal safe resection remains the clinical standard for GBM, as extent of resection has been repeatedly associated with improved survival in GBM cohorts and meta-analyses (104). The value of spatial immune mapping lies instead in guiding immune-aware sampling, postoperative immune risk stratification, and rational selection of local immunotherapeutic strategies. Because cytotoxic lymphocyte states may differ across the enhancing tumor core, perinecrotic regions, perivascular compartments, and the FLAIR-defined infiltrative edge, a single specimen from the enhancing core may misrepresent the patient-specific Tpex: Tex balance, NK-cell abundance, and myeloid suppressive architecture (15, 24, 25).

Future surgical and translational studies should therefore integrate image-guided, multi-regional sampling with immune profiling. When surgically feasible, sampled regions should include the enhancing core, necrotic or perinecrotic tissue, contrast-enhancing margin, FLAIR/infiltrative edge, and regions suspected to contain perivascular or immune-cell-rich niches, together with paired blood and, when clinically appropriate, paired CSF. This strategy is supported by anatomic transcriptional and single-cell studies showing that GBM molecular and cellular states vary across histologically and radiographically distinct tumor regions, including the infiltrative or migrating front (15, 24). Coupling these spatially annotated specimens with scRNA-seq or snRNA-seq, TCR sequencing, spatial transcriptomics, and multiplexed protein imaging would help determine whether Tpex-like cells are locally maintained, peripherally recruited, or spatially excluded in individual GBM tumors. In practical terms, spatial immune maps should guide what to sample and how to treat the postoperative cavity, not what tumor-bearing tissue should be intentionally left behind.

Spatial niche information may also inform local drug delivery. Putative perivascular or APC-rich niches that preserve Tpex-like cells could be leveraged for local checkpoint, cytokine, vaccine, or epigenetic modulation if validated in GBM, whereas perinecrotic myeloid-rich regions may require myeloid reprogramming or metabolic intervention. Clinically feasible delivery platforms include resection cavity-based biomaterials, intracavitary or intraventricular CAR-T/CAR-NK administration, Ommaya reservoir-based delivery, convection-enhanced delivery, and perioperative release of checkpoint-modulating, cytokine-modulating, or epigenetic agents (92, 105–107). These approaches may partially bypass systemic BBB/BTB constraints while enabling local pharmacodynamic assessment of immune remodeling in the postoperative tumor bed.

These spatially informed neurosurgical strategies, when integrated with the single-cell and epigenetic insights reviewed throughout this article, form the translational backbone of a precision immunotherapy framework for GBM.

## 8 Conclusions and future perspectives

This review has synthesized current single-cell and spatial multiomics evidence to construct a spatially and cellularly resolved framework for understanding cytotoxic lymphocyte heterogeneity and dysfunction in GBM. Three key insights emerge from this synthesis. First, GBM harbors a highly heterogeneous landscape of cytotoxic CD8^+^ T cell and NK cell subpopulations that differ mechanistically in their exhaustion pathways. T cell exhaustion is driven by TOX/NR4A-mediated epigenetic locking through chronic TCR stimulation, whereas NK cell dysfunction reflects receptor-ligand imbalance, tumor-side ligand silencing, and metabolic-epigenetic crosstalk unique to the CNS environment. Second, these cytotoxic subpopulations are spatially segregated within distinct tumor niches: Tpex cells are maintained in the perivascular zone, terminally exhausted T cells co-localize with suppressive TAMs in the perinecrotic niche, and NK cells are relatively enriched at the infiltrative edge. This spatial organization cannot be discerned from bulk transcriptomics and has direct implications for the accessibility and efficacy of immunotherapy. Third, CNS-specific features of GBM—including BBB restriction, Piezo1-driven mechanosensory Tpex depletion, IDH-mutation-driven NKG2D ligand silencing, and a TLS-poor immune landscape—impose unique constraints on cytotoxic lymphocyte function that differentiate GBM from other solid tumors and necessitate GBM-tailored therapeutic approaches.

Translating the insights from single-cell and spatial multiomics into clinical practice necessitates a paradigm shift toward spatially informed and personalized immunotherapy. Future therapeutic frameworks must integrate spatial mapping to guide region-specific interventions: perivascular niches enriched with TCF-1^+^ progenitor-exhausted T cells (Tpex) may be ideal targets for local checkpoint blockade or epigenetic priming to preserve the stem-like reservoir, whereas perinecrotic zones dominated by myeloid suppression may require metabolic reprogramming or macrophage repolarization prior to T cell-directed therapy. Furthermore, the integration of multi-omics data—spanning genomic, transcriptomic, and spatial proteomic layers—holds the key to patient stratification. By correlating specific spatial immune architectures (e.g., Tpex spatial distribution, NK cell exclusion patterns) with treatment response, we can move beyond the current “one-size-fits-all” approach to develop personalized combination regimens. This includes leveraging intraoperative spatial profiling to dynamically select between T cell-centric, NK cell-centric, or myeloid-targeted therapies based on the unique immune topology of each patient’s tumor, thereby maximizing therapeutic efficacy while minimizing spatial immune escape.

Critically, the immunological principles and therapeutic vulnerabilities of GBM must be stratified by IDH1 mutation status and transcriptional subtype, as they define distinct immune evasion mechanisms. IDH1-mutant gliomas are characterized by a 2-HG-driven hypermethylated landscape (G-CIMP) that epigenetically silences NKG2D ligands (ULBP1/3), resulting in profound NK cell immune evasion; thus, epigenetic reprogramming is the primary therapeutic imperative for this subtype. In contrast, IDH1-wildtype GBM exhibits a more heterogeneous and often highly myeloid-inflamed microenvironment dominated by metabolic competition (e.g., lactate) and spatial exclusion. Furthermore, molecular subtyping reveals distinct immune architectures: Mesenchymal (MES)-like tumors, defined by pronounced TGF-β signaling and a dominant immunosuppressive TAM population, are prime candidates for myeloid-targeted therapies; Classical (CL)-like tumors, driven by EGFR alterations, display distinct antigenicity suitable for targeted antibody therapies (e.g., Cetuximab) combined with NKG2A blockade to potentiate ADCC; and Proneural (PN)-like tumors, often PDGFRA-driven, typically exhibit a less inflamed “cold” microenvironment but may harbor specific antigens amenable to CAR-T/NK targeting. This stratification necessitates a paradigm shift from “one-size-fits-all” immunotherapy to a molecularly guided precision approach.

Looking forward, four research priorities emerge as particularly critical. The first is longitudinal multiomics tracking of cytotoxic lymphocyte subpopulation dynamics from diagnosis through recurrence and in response to immunotherapy, using paired pre- and post-treatment surgical specimens. Such datasets would transform our understanding of acquired immune resistance in GBM. The second priority is the development of GBM-specific organoid and cerebral organoid-tumor fusion models that recapitulate the CNS immune microenvironment—including its unique mechanical properties (Piezo1 activation), metabolic landscape (IDO, lactate, 2-HG), and cellular composition (microglia, astrocytes, BBB)—enabling mechanistic dissection of cytotoxic lymphocyte dysfunction in a system that preserves the CNS context absent from conventional tumor spheroid models. The third priority is the clinical integration of rapid intraoperative spatial immune profiling into GBM surgical workflows, leveraging the neurosurgical tissue access window to enable real-time immunotherapy stratification—a paradigm shift from one-size-fits-all to spatially personalized GBM immunotherapy. A fourth priority is systematic integration of spatial proteomics, scATAC-seq/multiome, and lineage tracing into GBM cytotoxic lymphocyte research, to move beyond descriptive transcriptomic atlases toward mechanistically resolved, epigenetically validated, and clonally trackable models of immune failure.

In summary, the convergence of single-cell transcriptomics, spatial multiomics, and mechanistic epigenetic biology is illuminating GBM immune failure with unprecedented resolution. The therapeutic insights emerging from this spatial and cellular dissection—epigenetic Tpex expansion, NKG2A/TIGIT dual NK cell reactivation, and intraoperative immune profiling—represent a new generation of neurosurgically actionable immunotherapy strategies that may finally overcome the profound barriers that have limited cytotoxic lymphocyte-directed therapies in GBM.
